# Passage number affects differentiation of sensory neurons from human induced pluripotent stem cells

**DOI:** 10.1038/s41598-022-19018-6

**Published:** 2022-09-23

**Authors:** Erica L. Cantor, Fei Shen, Guanglong Jiang, Zhiyong Tan, Geneva M. Cunningham, Xi Wu, Santosh Philips, Bryan P. Schneider

**Affiliations:** 1 Hematology/Oncology, Indiana University School of Medicine, Indianapolis, IN USA; 2 Medical & Molecular Genetics, Indiana University School of Medicine, Indianapolis, IN USA; 3 Pharmacology & Toxicology, Stark Neurosciences Research Institute, Indiana University School of Medicine, Indianapolis, IN USA; 4 Clinical Pharmacology, Indiana University School of Medicine, Indianapolis, IN USA

**Keywords:** Neurological models, Stem-cell differentiation

## Abstract

Induced pluripotent stem cells (iPSCs) are a valuable resource for neurological disease-modeling and drug discovery due to their ability to differentiate into neurons reflecting the genetics of the patient from which they are derived. iPSC-derived cultures, however, are highly variable due to heterogeneity in culture conditions. We investigated the effect of passage number on iPSC differentiation to optimize the generation of sensory neurons (iPSC-dSNs). Three iPSC lines reprogrammed from the peripheral blood of three donors were differentiated into iPSC-dSNs at passage numbers within each of the following ranges: low (5–10), intermediate (20–26), and high (30–38). Morphology and pluripotency of the parent iPSCs were assessed prior to differentiation. iPSC-dSNs were evaluated based on electrophysiological properties and expression of key neuronal markers. All iPSC lines displayed similar morphology and were similarly pluripotent across passage numbers. However, the expression levels of neuronal markers and sodium channel function analyses indicated that iPSC-dSNs differentiated from low passage numbers better recapitulated the sensory neuron phenotype than those differentiated from intermediate or high passage numbers. Our results demonstrate that lower passage numbers may be better suited for differentiation into peripheral sensory neurons.

## Introduction

Induced pluripotent stem cells (iPSCs) are a valuable resource for neurological disease-modeling and drug discovery, largely due to their relative accessibility and capacity to differentiate into neurons with the same genetic background as the patient from which they are derived^1–6^. Studies on neurological disease usually have limited accessibility to human nerve tissue due to the potential for significant pain and permanent nerve damage during biopsy. Thus far, while animal models and immortalized primary neuron culture models have been used, there remain significant translational limitations. Thus, the value of iPSCs comes from their ability to be differentiated into cell types that may be otherwise inaccessible for study. iPSC-derived sensory neurons (iPSC-dSNs), for example, can be utilized in high-throughput assays to study diseases affecting the peripheral nervous system, such as peripheral neuropathy, and for the discovery of new drug targets. Reliable differentiation of sensory neurons from patient-specific somatic tissue has the potential to serve as a valuable resource for studying disparities in treatment response and disease progression based on germline genetic factors related to sex, age, or ancestry.

Current reprogramming methods that allow for the derivation of human iPSCs from various somatic tissue types, such as keratinocytes, fibroblasts, and peripheral blood, have greatly advanced neuroscience^7–17^. However, the implementation of iPSCs as a model is not without limitation. Several studies have demonstrated significant variability in the behavior of reprogrammed iPSC lines^18–24^, and specifically in the differentiation of sensory neurons from iPSCs^25–30^. Among various factors that may contribute to this variability, the impact of iPSC passage number is not clearly understood.

In this study, we examined the effect of iPSC passage number on sensory neuronal differentiation using three lines reprogrammed from the peripheral blood of three donors. We assessed the morphology and pluripotency of each iPSC line at each of three (low, intermediate, and high) passage numbers before differentiation. We then assessed morphology, gene expression, and electrophysiological properties in the resultant iPSC-dSNs to gain a better understanding of the effect of passage number on the derivation of mature, functional sensory neurons.

## Results

### Passage number does not affect morphology or pluripotency of iPSCs

**Figure 1:**
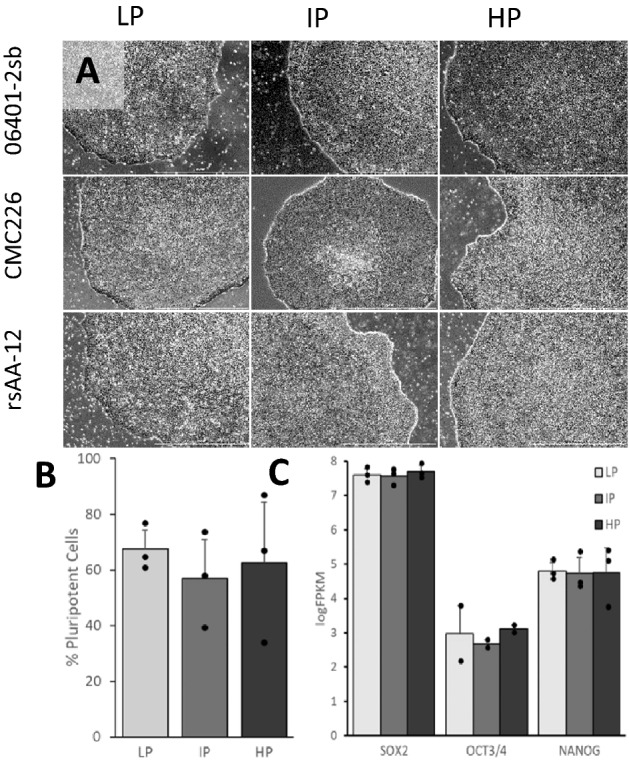
Morphology and pluripotency of the three iPSC lines (06401-2sb, CMC226, rsAA-12) at different passage numbers (low, intermediate, and high). (**A**) Phase-contrast images of three iPSC cell lines at different passage numbers. Scale bar = 1000 microns. (**B**) The average proportion of pluripotent cells in iPSCs at different passage numbers based on measurements of the expression of Sox2, Oct3/4, and Nanog using flow cytometry. (**C**) The expression level of *SOX2*, *OCT3/4*, and *NANOG* in iPSCs at different passage numbers based on RNA-seq. LP = low-passage, IP = intermediate-passage, HP = high-passage.

The morphology and pluripotency of three different iPSC lines (06401-2sb, CMC226, and rsAA-12), each at low (LP), intermediate (IP), and high passage (HP) numbers, were compared. Phase-contrast images demonstrated the expected morphology of flat, well-defined cell colonies in iPSCs of all passage numbers (Fig. 1A). To assess the effect of passage number on the pluripotency of iPSCs, we compared the expression levels of pluripotency markers Sox2, Oct3/4, and Nanog in iPSCs by flow cytometry to determine the proportion of pluripotent cells. Our results indicated that there was no significant difference in the proportion of pluripotent cells across passage number, with an average of 67.5%, 56.9%, and 62.6% of iPSCs expressing all three factors at low, intermediate, and high passage numbers, respectively (Fig. 1B). We further assessed the expression of *SOX2*, *OCT3/4*, and *NANOG* by RNA-seq and the results were consistent; expression of these markers was not significantly different across passage numbers (Fig. 1C). These data indicate that passage number did not affect iPSC morphology or pluripotency.

### Passage number has no effect on morphology and expression of peripherin and βIII-tubulin in iPSC-dSNs

**Figure 2:**
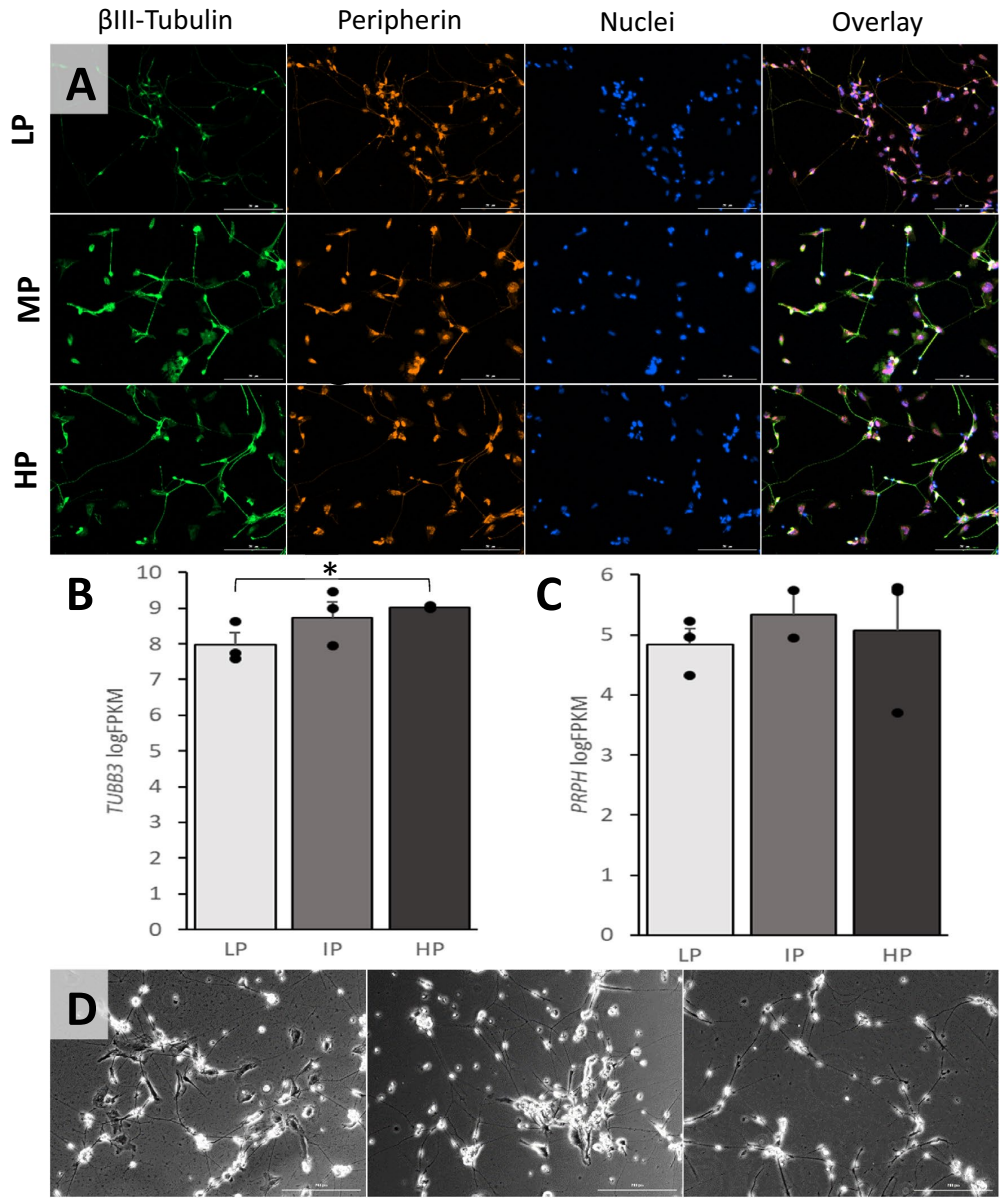
The morphology and expression of neuronal markers peripherin and βIII-tubulin in iPSC-dSNs differentiated from iPSCs at each of low, intermediate, and high passage numbers. (**A**) Representative immunofluorescent images of neurons stained for neuronal markers peripherin and βIII-tubulin. Scale bar = 200 microns. (**B**) Average expression levels of *TUBB3* in iPSC-dSNs derived from iPSCs with different passage numbers by RNA-seq. (**C**) Average expression levels of *PRPH* in iPSC-dSNs derived from iPSCs with different passage numbers by RNA-seq. (**D**) Representative phase-contrast images of iPSC-dSNs derived from iPSCs with different passage numbers. Scale bar = 200 microns. LP = low-passage, IP = intermediate-passage, HP = high-passage; *FDR = 0.005.

iPSC-dSNs derived from three different iPSC lines at different passage numbers were co-stained for neuronal markers peripherin and βIII-tubulin at day 33 post-induction. Immunofluorescent images of these neurons presented visually similar staining patterns (Fig. 2A). There were also no marked differences in the expression levels of *PRPH* (peripherin) or *TUBB3* (βIII-tubulin) across passage numbers based on RNA-seq, except for a slightly higher expression of *TUBB3* in the HP lines than in the LP lines (9.03 vs. 7.98; FDR = 0.005) (Fig. 2B,C). Phase-contrast images revealed similar morphology of iPSC-dSNs across starting iPSC passage numbers (Fig. 2D). Taken together, starting iPSC passage number did not significantly affect the expression of neuronal markers peripherin and βIII-tubulin or neuronal morphology in iPSC-dSNs.

### LP iPSCs produce more mature, sensory-like neurons

**Figure 3:**
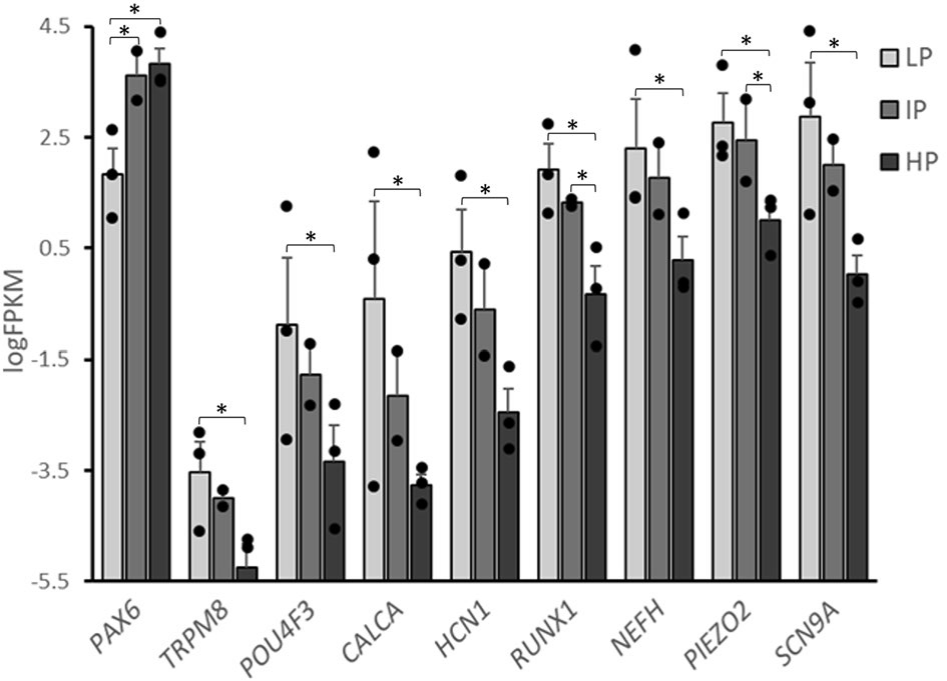
Comparison of average expression levels of neuronal marker genes at day 33 post-induction in three iPSC-dSN lines induced at three different iPSC passage numbers. LP = low-passage, IP = intermediate-passage, HP = high-passage; * FDR < 0.05.

To assess the maturity and sensory properties of the differentiated neurons, we examined the expression levels of the immature neuron marker gene *PAX6* and several mature sensory neuron markers (Fig. 3)*.* Among the three passage number groups from three cell lines, LP iPSC-dSNs had a significantly lower expression of *PAX6* compared to both IP (FDR = 0.006) and HP (FDR = 1.1 × 10^–4^) iPSC-dSNs at day 33 post-induction. These data indicate that the LP iPSC-dSNs achieved greater maturity at this timepoint. More importantly, LP iPSC-dSNs had the highest expression levels of several key mature sensory neuron marker genes at day 33 post-induction, including *TRPM8, POU4F3, CALCA, HCN1, RUNX1*, *NEFH, PIEZO2, SCN9A,* and *RUNX1,* among the three passage groups; the differences in the expression of these marker genes between LP and HP iPSC-dSNs were statistically significant, with FDRs ranging from 0.04 to 1.7 × 10^–5^, further suggesting greater maturity of the LP iPSC-dSNs. These results indicate that the LP iPSC-dSNs better recapitulated the desired sensory neuron phenotype.

### LP iPSCs produce larger, more electrophysiologically mature neurons

**Figure 4:**
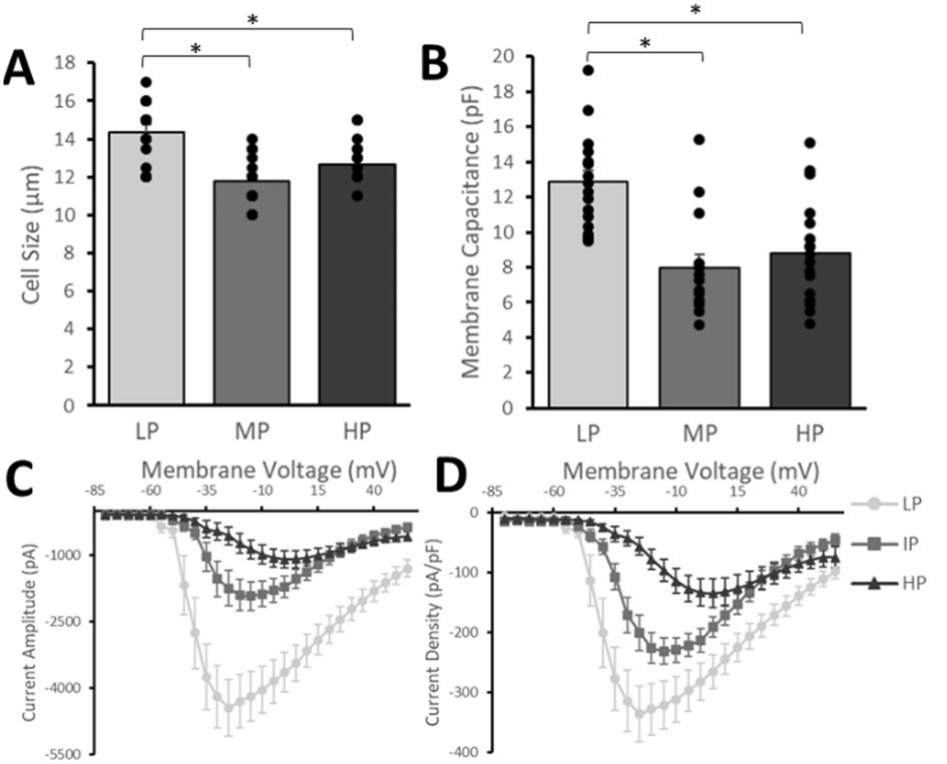
Electrophysiological assessment of iPSC-dSNs differentiated from iPSCs at different passage numbers. (**A**) Average cell size, (**B**) average membrane capacitance, and (**C**) average sodium current amplitude and (**D**) density from whole-cell patch-clamp recordings. Experiments were performed using 16, 14, and 18 iPSC-dSNs of low-, intermediate-, and high-passage, respectively. LP = low-passage, IP = intermediate-passage, HP = high-passage; *p-value < 0.05.

Electrophysiological characterization of the iPSC-dSNs derived from different passage numbers demonstrated that LP iPSC-dSNs had a significantly greater average cell size (14.38 ± 1.60 µm) compared to IP (11.75 ± 1.38 µm) and HP (12.67 ± 1.16 µm) iPSC-dSNs (Fig. 4A). The membrane capacitance of LP iPSC-dSNs was 1.6- and 1.5-fold higher than that of IP and HP iPSC-dSNs, respectively (Fig. 4B). LP iPSC-dSNs also had significantly higher sodium current amplitudes and density at some or all voltages from − 40 to 55 mV than IP or HP iPSC-dSNs (Fig. 4C,D). These results suggest that LP iPSC-dSNs demonstrated superior electrophysiological maturity. IP iPSC-dSNs also had significantly higher sodium current amplitudes and density than HP iPSC-dSNs at some or all voltages from − 35 to 0 mV, suggesting that IP iPSC-dSNs, though inferior to LP iPSC-dSNs, also displayed better electrophysiological maturity compared to HP iPSC-dSNs.

## Discussion

Several studies have demonstrated significant variability in neuronal differentiation from iPSCs^25–30^. Factors related to iPSC culture, such as passage number, have been shown to be significant contributors to this variability^2,28,31–37^. Some have suggested that differences in differentiation efficiency may be due to an “epigenetic memory” of the tissue of origin^19,20,34,38–45^. These genetic remnants are thought to be more prominent at lower passages and might be reduced by extended passaging of an iPSC line^33,42,46–49^, however, others demonstrated that increased passaging could introduce greater genomic instability and structural variations^31,32,50–53^. Tang et al.^54^ demonstrated that the differentiation of low-passage human neural progenitor cells resulted in a higher percentage of neurons, while higher-passage NPCs gave rise to more glial cells. This would suggest that the passage number of human pluripotent cells on differentiation may be more complex, and the optimization of passage number is necessary depending on the specific endpoint phenotype desired. Our study assessed and compared the quality of peripheral sensory neurons differentiated from iPSCs at different passage numbers to optimize the generation of neurons that better recapitulate the desired peripheral sensory phenotype.

We assessed the relative maturity of iPSC-dSNs based on the expression of several key neuronal markers. *PAX6* encodes a key transcription factor in the development of neural tissues and is a marker for neural progenitor cells. During in vivo neuronal differentiation, *PAX6* is known to be upregulated shortly after induction and then downregulated as the neurons mature. In our study, LP iPSC-dSNs expressed significantly lower levels of *PAX6* at day 33 post-induction as compared to IP and HP iPSC-dSNs, suggesting that the LP cells may have achieved greater maturity by this timepoint. More importantly, LP iPSC-DSNs exhibited higher expression levels of several marker genes for mature sensory neurons, including genes encoding transcription factors (*RUNX1* and *POU4F3),* ion channels (*PIEZO2, TRMP8, HCN1,* and *SCN9A*,) cytoskeletal components (*NEFH)*, and the calcitonin gene-related peptide (CGRP)-producing gene, *CALCA*. Overall, these results strongly indicate that LP iPSCs are more efficient in generating mature sensory neurons. Although iPSC-derived neurons are known for exhibiting a more immature neuron-like phenotype^55–58^, our results suggest that this limitation may be at least partially overcome by differentiating iPSCs at lower passage numbers (< 10) and avoiding differentiation at high passage numbers (> 30).

Importantly, however, our study did not indicate that parent iPSC morphology, pluripotency, iPSC-dSN morphology, or expression of the common neuronal markers βIII-tubulin and peripherin, were associated with passage number. We also did not observe any marked differences in morphology or the expression of neuronal markers βIII-tubulin and peripherin in iPSC-dSNs from different passage number groups, indicating that iPSC passage number did not affect the expression of these neuronal markers in the differentiated sensory neurons. Thus, it is not clear whether βIII-tubulin and peripherin are simply not effective determinants of neuronal quality or whether there is a more complicated interplay. While our study focused on the expression of βIII-tubulin and peripherin neuronal markers, another study conducted by Tagliafierro et al.^59^ evaluated the expression of Lamin proteins in iPSC-derived dopaminergic and cholinergic neurons in order to recapitulate aging processes in the neuronal nuclear architecture.

Generating neurons that are electrophysiologically mature is important for disease-modeling, as many diseases alter electrophysiological properties. Using whole-cell patch-clamp recordings, this study demonstrated that differentiating iPSCs at low passages resulted in sensory neurons with a larger soma size and corresponding greater membrane capacitance. While the biological significance of cell size and capacitance differences are highly debated and difficult to ascertain, they may be important depending on the intended downstream assays using iPSC-dSNs. LP iPSC-dSNs also exhibited greater sodium current amplitude and density, suggesting a lower action potential threshold and greater action potential amplitude, and therefore greater electrophysiological maturity. This supports the superior maturity of the LP cells, along with our expression findings. Similarly, IP iPSC-dSNs exhibited larger sodium currents and presumably greater electrophysiological maturity compared to HP iPSC-dSNs.

Previously, little work has been done to examine the specific effect of iPSC passage number on the differentiation of sensory neurons. In this study, we have demonstrated a simple, effective method for differentiating sensory neurons from iPSCs reprogrammed from human peripheral blood mononuclear cells and have assessed the impact of iPSC passage number on the heterogeneity of these neurons. Here, we demonstrated that, in an iPSC-derived peripheral sensory neuron model, inducing neuronal differentiation at lower passages (< 10) will more consistently produce neurons that achieve greater maturity and electrophysiological properties by day 33. Higher passage iPSCs likely become vulnerable to greater genomic instability, resulting in less efficient differentiation. These findings may be utilized to improve the reliability of disease-modeling and drug discovery related to disorders of the peripheral nervous system. It is important to note that significant variability exists in iPSC-dSN culture with regards to the specific reprogramming and differentiation methods utilized. Therefore, though it may be appropriate to apply the findings of this study more broadly, future studies, such as analyses on the percent of cells successfully differentiated to neurons, differential transcriptomics, or protein-level corroboration of a greater number of genes, are warranted to provide more insight into the underlying mechanisms that contribute to the variability in differentiation of sensory neurons seen across iPSC passage numbers.

## Methods

### Generation of iPSCs

**Figure 5:**
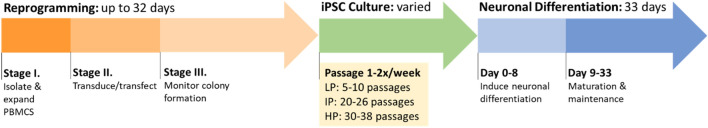
Timeline of reprogramming iPSCs from peripheral blood and subsequent neuronal differentiation.

All donor samples were obtained with informed, written consent under the approval of the Indiana University Institutional Review Boards (IRB) 1106005767 and all experiments were performed in accordance with the relevant guidelines and regulations. Peripheral blood was collected from three individual donors separately and mononuclear cells (PBMCs) were isolated following either the Erythroid Progenitor Reprogramming Kit (STEMCELL Technologies Inc.) protocol (cell lines 06401-2sb and rsAA-12) or using the BD Vacutainer® CPT™ Tube with Sodium Citrate (BD Biosciences) (cell line CMC226) (Fig. 5).

PBMCs isolated with the Erythroid Progenitor Reprogramming Kit continued to be reprogrammed using this protocol. Briefly, erythroid progenitor cells were expanded for seven days in StemSpan™ SFEM II supplemented with 1% StemSpan™ Erythroid Expansion Supplement (STEMCELL Technologies Inc.) before being transfected with Epi5™ episomal vectors (Invitrogen™) containing five reprogramming factors (Oct4, Sox2, Lin28, Klf4, and L-Myc) via nucleofection. Cells were then transitioned into ReproTeSR™ medium (STEMCELL Technologies Inc.) over seven days. After completing the transition, the cells were maintained in ReproTeSR™ medium (STEMCELL Technologies Inc.) and monitored for up to 18 more days until the formation of iPSC colonies.

PBMCs isolated using the BD Vacutainer® CPT™ tube were reprogrammed using the CytoTune™-iPS 2.0 Sendai Reprogramming Kit (Invitrogen™). Briefly, PBMCs were expanded in StemPro™-34 medium (STEMCELL Technologies Inc.) supplemented with 2 mM L-Glutamine and cytokines. After four days of expansion, the PBMCs were transduced with Sendai virus vectors containing the four Yamanaka factors, Oct4, Sox2, Klf4, and c-Myc. Viruses were removed the following day and the transduced cells were maintained on Matrigel-coated plates for an additional seven days, after which they were transitioned into mTeSR™ Plus (STEMCELL Technologies, Inc.) and maintained for up to 20 more days while awaiting the formation of iPSC colonies.

iPSC colonies were then allowed to grow until reaching an appropriate size for selection and transfer. iPSC colonies were then maintained in mTeSR™ Plus medium and tested for pluripotency as described below. Normal karyotypes (46,XX) were confirmed for each line using G-banded cytogenetic analysis.

### Maintenance of iPSCs

iPSCs were maintained in mTeSR™ Plus medium on Matrigel-coated plates by a single researcher in order to reduce experimental variability. Medium was replaced daily, and cultures were frequently observed for spontaneous differentiation. Any observed differentiation was removed manually. Each line was passaged one to two times per week using Dispase (1 U/mL in DMEM/F-12; STEMCELL Technologies Inc.) until each target passage number (below) was reached (nine samples total; three cell lines with three target passage numbers each). Low-passage cells were analyzed after 5–10 passages, intermediate-passage cells after 20–26 passages, and high-passage cells after 30–38 passages. The MycoAlert™ Mycoplasma Detection Kit (Lonza) was utilized to confirm the absence of contamination.

### Measurement of pluripotency of iPSC lines

The pluripotency of each line was tested via flow cytometry at each of the target passage numbers using the Human Pluripotent Stem Cell Transcription Factor Analysis Kit (BD Biosciences). The expression of three core pluripotency transcription factors (Oct3/4, Sox2, and Nanog) was examined graphically. Unstained and isotype controls were included with every sample.

### Neuronal differentiation

iPSCs from the three different cell lines were induced into sensory neuronal differentiation on Matrigel-coated 6-well plates at ~ 80% confluence at each of three different passage numbers (low, intermediate, and high), as described above. Differentiation into sensory neurons (iPSC-dSNs) was achieved via a previously published protocol^60^ and source information for all small molecules, growth factors, and antibodies used can be found in Table 1. Briefly, differentiation was induced using DMEM/F12 supplemented with KnockOut™ Serum Replacement (10%) (Gibco), LDN-193189 (0.3 µM), A83-01 (2 μM), CHIR 99021 (6 μM), RO4919097 (2 μM), SU 5402 (3 μM), and retinoic acid (0.3 μM). Cells were maintained in this induction medium for eight days, with complete medium replacement occurring every other day. On day nine post-induction, cells were dissociated with Accutase™ (STEMCELL Technologies Inc.). Cells were then strained through a 37 µm mesh and single-cell seeded onto new Matrigel-coated 6-well plates (6 × 10^5 cells/well) in a maintenance medium consisting of Neurobasal™ Plus medium supplemented with B-27 Plus (1%) (Gibco), neurotrophin-3 (10 ng/mL), brain-derived neurotrophic factor (20 ng/mL), nerve growth factor (20 ng/mL), and glial cell line-derived growth factor (20 ng/mL). This maintenance medium was replaced every other day and cells were allowed to mature for up to an additional 24 days.

**Table 1:** Source information for the materials used in neuronal differentiation and immunofluorescence.

Product	Manufacturer	Catalog number
**Small molecules**		
StemMACS™ LDN-193189	Miltenyi Biotec	130–103-925
StemMACS™ A83-01	Miltenyi Biotec	130–105-333
CHIR 99021	BioVision Inc	1677–25
RO4919097	Selleck Chemical LLC	S157510MG
SU 5407	Tocris	3300
Retinoic acid	Sigma Aldrich	R2625
**Growth factors**		
Human NT-3 Recombinant Protein	Gibco	PHC7036
Human beta-NGF Recombinant Protein	Gibco	PHG0126
Human GDNF Recombinant Protein	Gibco	PHC7045
Human BDNF Recombinant Protein	Gibco	PHC7074
**Immunofluorescence antibodies**		
Anti-Peripherin antibody	Abcam	ab4666
Anti-beta III Tubulin antibody	Abcam	ab78078

### Phase-contrast imaging

Phase-contrast images of the parent iPSCs and differentiated iPSC-dSNs were captured using the Lionheart FX Automated Microscope (BioTek Instruments, Inc.). Focus and exposure settings were adjusted for optimal visualization. Representative images of the morphology of the three cell lines were captured at either 4X (iPSCs) or 10X (iPSC-dSNs) magnification at low, intermediate, and high passage numbers for visual comparison to assess for any differences in morphology across passage numbers.

### Immunofluorescence imaging

On day 28 post-induction, iPSC-dSNs were dissociated using Accutase™ (STEMCELL Technologies, Inc.) and single-cell seeded onto Matrigel-coated 12-well plates at 1.5 × 10^5^ cells/well. The cells were then fixed with paraformaldehyde (4%) on day 33 post-induction and were subsequently stained for immunofluorescence imaging with antibodies against peripherin (1:1000; Abcam) and βIII-tubulin (1:1000; Abcam). Nuclei were stained with NucBlue™ Fixed Cell ReadyProbes™ Reagent (1:1000; Invitrogen™). All staining was carried out by the same researcher in order to reduce variability. Images were captured at 10X magnification using the Lionheart FX Automated Microscope. Representative images for each of the cell lines at different passage numbers were compared visually to assess for any differences in the distribution of the stains or co-staining patterns, as well as morphology.

### Electrophysiology

On day 28 post-induction, iPSC-dSNs derived from iPSC line 06401-2sb at each of three passage numbers were single-cell seeded onto 5 × 5 mm Matrigel-coated plastic coverslips at a density of 1.0–1.5 × 10^5^ cells/coverslip. Cell density was adjusted within this range as necessary to achieve proper spacing for patch-clamping. On day 33 post-induction, whole-cell patch-clamp recordings were conducted in voltage-clamp mode at room temperature as previously reported^61,62^. An Axopatch 200B patch-clamp amplifier (Molecular Devices) was utilized and data were acquired using the pClamp (v8.0) software (Molecular Devices). Borosilicate glass capillaries were used to construct fire-polished electrodes (1.5–2.5 MΩ). The standard electrode solution contained CsF (140 mM), NaCl (10 mM), EGTA (1.1 mM), and HEPES (10 mM, pH 7.3). The standard extracellular bathing solution consisted of NaCl (130 mM), TEA chloride (30 mM), MgCl_2_ (1 mM), KCl (3 mM), CaCl_2_ (1 mM), CdCl_2_ (0.05 mM), HEPES (10 mM), and D-glucose (10 mM; pH 7.3).

### RNA-sequencing

Total RNA was extracted from cells on days 0 (iPSCs) and 33 (iPSC-dSNs) post-induction using the RNeasy Mini Kit (QIAGEN). Three cell lines were used at three different passage numbers (low, middle, and high), for a total of 9 iPSC samples and 9 iPSC-dSN samples. Quality and quantity were evaluated with the 2100 Bioanalyzer (Agilent Technologies); the average RIN number across all samples was 9.71. 100 ng of RNA was used for cDNA library preparation, including mRNA purification/enrichment, RNA fragmentation, cDNA synthesis, ligation of index adaptors, and amplification, following the KAPA mRNA Hyper Prep Ki Technical Data Sheet, KR1352–v4.17 (Roche Corporate). The resulting indexed libraries were quantified and assessed for quality using a Qubit™ Fluorometer (Invitrogen™) and the 2100 Bioanalyzer. Multiple libraries were pooled in equal molarity and then were denatured and neutralized. The libraries were loaded onto the NovaSeq 6000 (Illumina, Inc.) sequencer for 100 base-paired sequencing at a concentration of 300 pM. Approximately 30 million reads were generated per library. The quality of the sequencing data was assessed using FastQC (Babraham Bioinformatics) and all sequenced libraries were mapped to the human genome (hg38) with STAR RNA-seq aligner using the parameter “—outSAMmapqUnique 60.” Bamutils (from ngsutils) was utilized to assess the reads distribution across the genome. Uniquely mapped reads were assigned to hg38 refGene genes using featureCounts (from subread) with the parameters “-s 2 -p -q 10”. MultiQC (v1.9) was utilized to summarize quality control results for sequencing and mapping; genes with a CPM > 0.2 in less than 3 samples were removed from further analyses.

### Statistical Analyses

Differential expression analysis of pluripotency genes (*NANOG*, *OCT3/4*, and *SOX2*), and neuron marker genes (*TUBB3*, *PRPH*, *PAX6, TRPM8, POU4F3, CALCA, HCN1, RUNX1*, *NEFH, PIEZO2,* and *SCN9A*) were conducted separately using three cell lines in each passage number group at day 33 post-induction with a likelihood ratio test using the edgeR package in R (v3.6.2), with the cell line and passage number as independent variables. The Benjamin-Hochberg procedure was used for multiple comparisons correction of the selected genes and a false discovery rate (FDR) less than 0.05 was considered statistically significant.

Raw electrophysiology data was averaged across all cells in each passage group and analyzed using GraphPad Prism v9.0.0. Differences in average cell size and membrane capacitance were assessed via one-way ANOVA. A post-hoc Tukey test was conducted to determine which specific passage number comparisons were statistically different. Average sodium current amplitude and density were assessed, and means were compared using a two-way mixed-model ANOVA and post-hoc Bonferroni test. Corrected p-values less than 0.05 were considered statistically significant. Patch-clamp data was obtained from 16, 14, and 18 cells of low, intermediate, and high passage, respectively.

## Data Availability

The corresponding author can provide the datasets utilized in this study on reasonable request. The raw gene expression data is available on Gene Expression Omnibus, GSE193571 (https://www.ncbi.nlm.nih.gov/geo/query/acc.cgi?acc=GSE193571).
